# Supplementary material for: A signal motif retains Arabidopsis ER-α-mannosidase I in the cis-Golgi and prevents enhanced glycoprotein ERAD
Source: Nat Commun. 2019 Aug 16;10:3701. doi: 10.1038/s41467-019-11686-9 (PMC6697737; doi:10.1038/s41467-019-11686-9)
Supplement: Supplementary file 1 — Supplementary Information [file 41467_2019_11686_MOESM1_ESM.pdf]

Supplementary Information

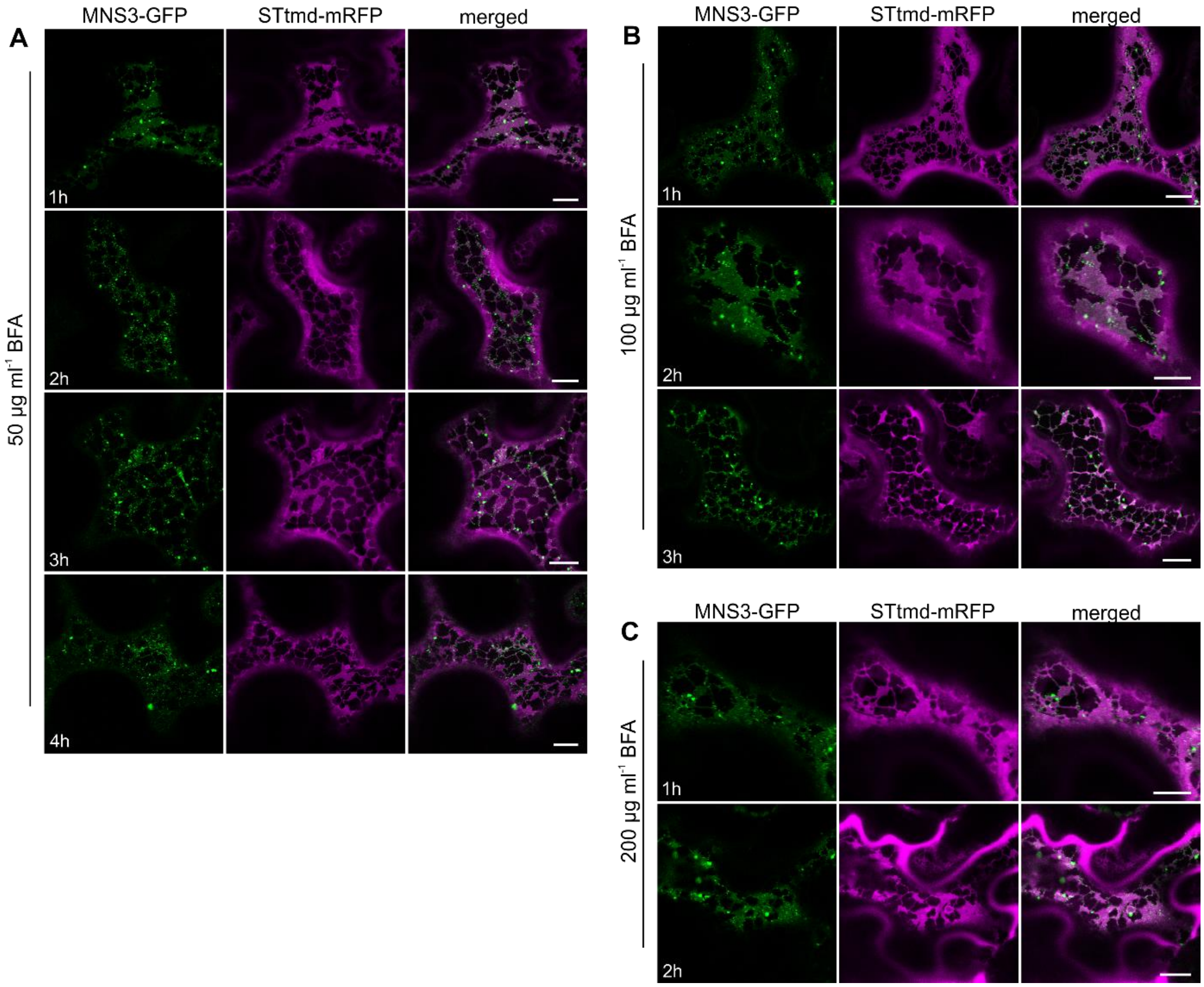

Supplementary Figure 1. Time-course treatment of MNS3-GFP with BFA using different concentrations. MNS3-GFP (green) was transiently co-expressed with STtmd-mRFP (magenta) in *N. benthamiana* leaf epidermal cells and observed 2 dpi on a confocal microscope. (A) Treatment of cells with 50 µg ml^{-1} BFA for 4 h. (B) Treatment of cells with 100 µg ml^{-1} BFA for 3 h. (C) Treatment of cells with 200 µg ml^{-1} BFA for 2 h. Scale bars = 10 µm.

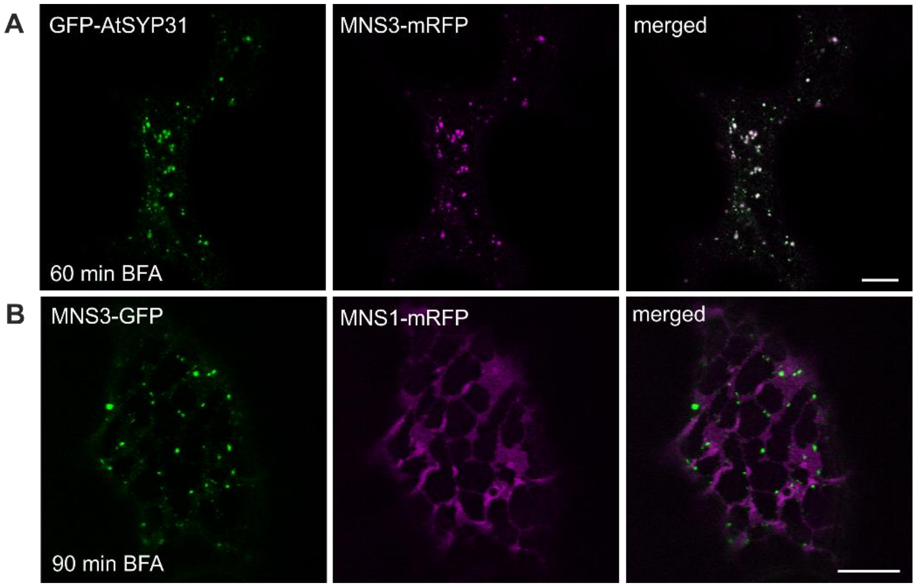

Supplementary Figure 2. Distinct *cis*-Golgi proteins show different BFA responses. Fluorescent protein fusions were transiently expressed in *N. benthamiana* leaf epidermal cells and observed 2 dpi on a confocal microscope. Images show the subcellular co-localization of (A) GFP-AtSYP31 (green) and MNS3-mRFP (magenta) after 60 min BFA, and (B) MNS3-GFP (green) and MNS1-mRFP (magenta) after 90 min BFA. Scale bars = 10 µm.

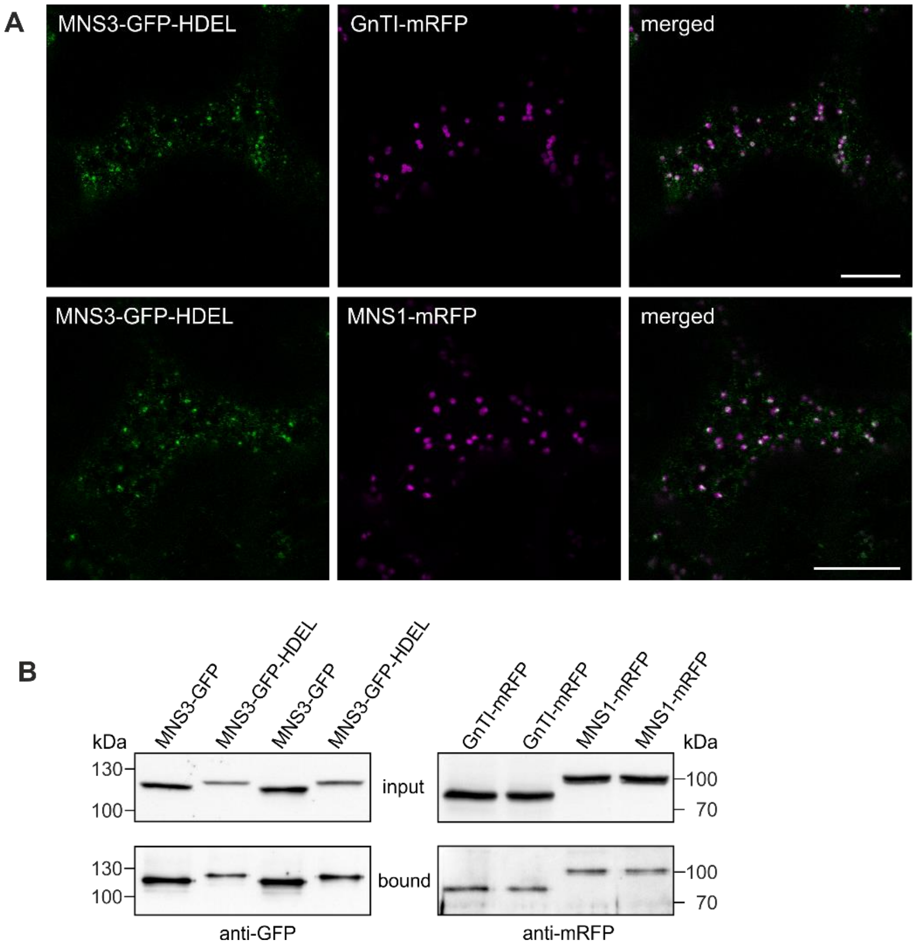

Supplementary Figure 3. Characterization of MNS3-GFP-HDEL. (A) Confocal images showing representative *N. benthamiana* leaf epidermal cells transiently expressing MNS3-GFP-HDEL (green) with the *cis*/medial-Golgi proteins GnTI-mRFP (magenta, upper panel) or MNS1-mRFP (magenta, lower panel). Images were acquired 2 dpi. Scale bars = 15 µm. (B) MNS3-GFP or MNS3-GFP-HDEL were transiently co-expressed with GnTI-mRFP and MNS1-mRFP, respectively, in *N. benthamiana* leaves. MNS3-GFP as well as MNS3-GFP-HDEL were purified using GFP-Trap beads. The GFP bait and co-purified proteins were analyzed by immunoblotting with antibodies against GFP and mRFP. “Input” denotes total protein extracts before incubation with GFP-coupled beads; “bound” denotes the immunoprecipitated fraction. Source data are provided as a Source Data file.

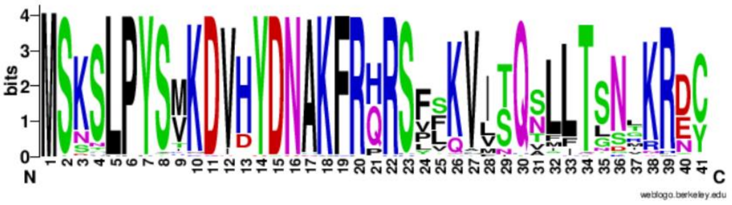

Supplementary Figure 4. Graphical representation of the conserved LPYS motif within multiple plant ER-α-mannosidases. Sequence logo of the consensus sequence from the alignment of the N-terminal 41 amino acids of the MNS3 cytoplasmic tail domain with similar plant protein sequences from the NCBI protein database. Source data are provided as a Source Data file.

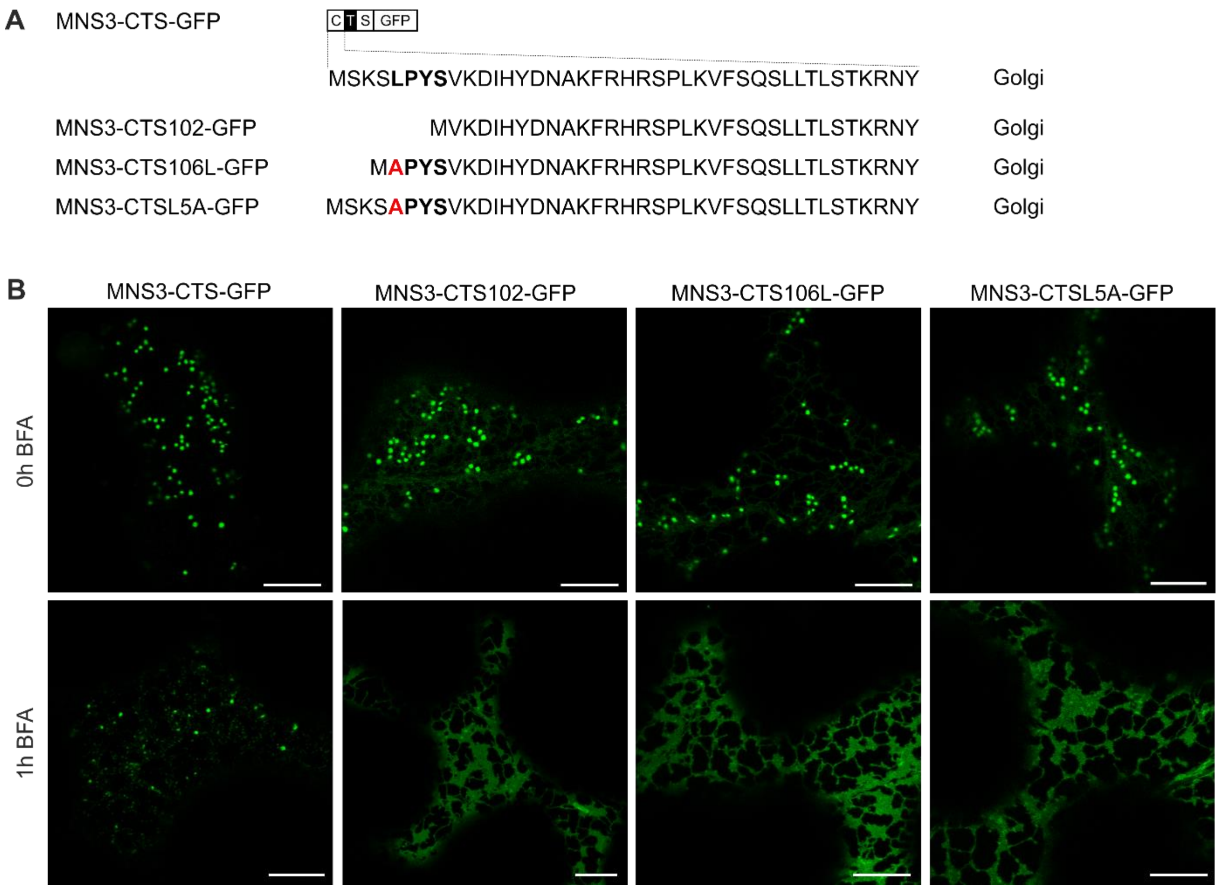

Supplementary Figure 5. Subcellular localization of MNS3 tail mutants before and after BFA treatment. (A) The modular organization of cytoplasmic-tail truncated and mutated constructs of the MNS3-CTS region fused to GFP and their subcellular localization in *N. benthamiana* leaf epidermal cells. (B) Confocal images of representative cells showing the subcellular localization of MNS3-CTS-GFP tail-mutated fusion proteins before and after 1h BFA treatment. Images were acquired 2 dpi. Scale bars = 10 µm.

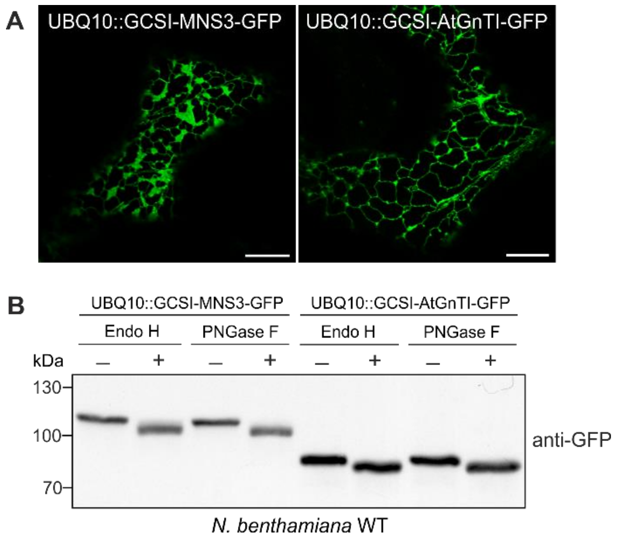

Supplementary Figure 6. Subcellular localization of chimeric GCSI-CTS fusion proteins. (A) Confocal images of UBQ10::GCSI-MNS3-GFP or UBQ10::GCSI-AtGnTI-GFP (catalytic domain of Arabidopsis GnTI) transiently expressed in *N. benthamiana* leaves. Images were acquired 2 dpi. Scale bars = 10 µm. (B) Crude protein extracts from leaves of *N. benthamiana* wildtype (WT) plants transiently expressing UBQ10::GCSI-MNS3-GFP or UBQ10::GCSI-AtGnTI-GFP were subjected to Endo H and PNGase F digestion followed by immunoblotting with anti-GFP antibodies. Source data are provided as a Source Data file.

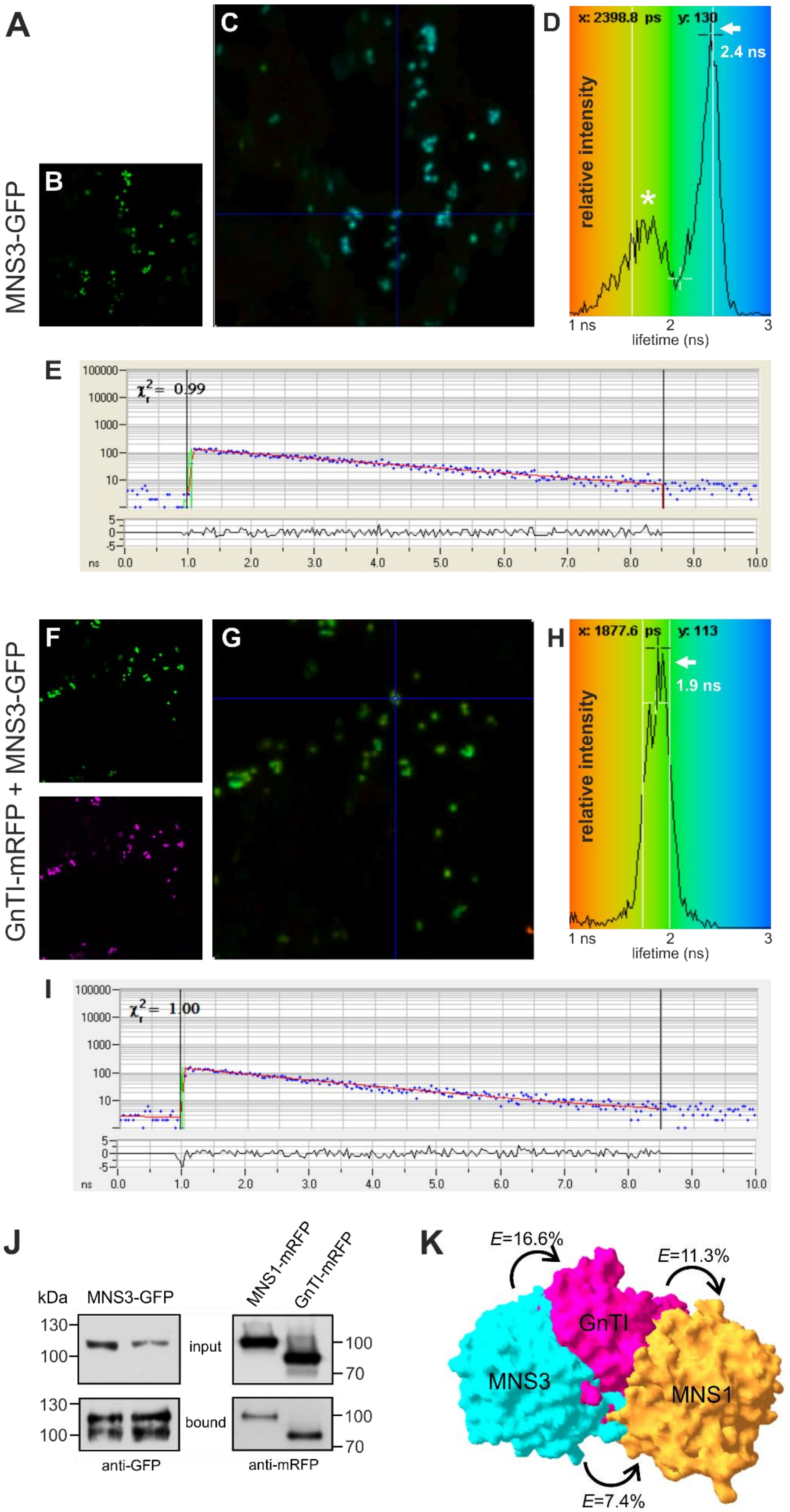

A
B
C
D
MNS3-GFP
x: 2398.8 ps y: 130
2.4 ns
relative intensity
*
1 ns
2
3
lifetime (ns)
E
χ_r^2 = 0.99
100000
10000
1000
100
10
5
0
-5
ns 0.0 1.0 2.0 3.0 4.0 5.0 6.0 7.0 8.0 9.0 10.0
F
G
H
GnTI-mRFP + MNS3-GFP
x: 1877.6 ps y: 113
1.9 ns
relative intensity
1 ns
2
3
lifetime (ns)
I
χ_r^2 = 1.00
100000
10000
1000
100
10
5
0
-5
ns 0.0 1.0 2.0 3.0 4.0 5.0 6.0 7.0 8.0 9.0 10.0
J
kDa
MNS3-GFP
MNS1-mRFP
GnTI-mRFP
130
100
input
100
70
130
100
bound
100
70
anti-GFP
anti-mRFP
K
E=16.6%
E=11.3%
GnTI
MNS3
MNS1
E=7.4%

Supplementary Figure 7. Protein-protein interactions of MNS3 in the Golgi apparatus. (A) *In vivo* FRET-FLIM analysis of MNS3. The donor protein MNS3-GFP was transiently expressed alone (B-E) or together with the acceptor protein GnTI-mRFP (F-I) in *N. tabacum* leaf epidermal cells and subjected to confocal imaging and two-photon FRET-FLIM (3 dpi). (B) and (F) Confocal images showing expression of MNS3-GFP (green) alone or with GnTI-mRFP (magenta). (C) and (G) Respective pseudo-colored lifetime maps showing the lifetime values for each point within the region of interest. (D) and (H) Distribution of GFP fluorescence lifetimes across the entire image, with blue shades representing higher lifetimes than green ones. (E) and (I) Representative decay curves of a single point with an optimal single exponential fit (binning factor of 2). This example of FRET-FLIM analysis shows that MNS3 interacts with GnTI, because the average lifetime value for the MNS3-GFP and GnTI-mRFP pair (1.99 ± 0.08 ns, n = 301) is lower than that for MNS3-GFP alone (2.38 ± 0.09 ns, n = 283). Mean ± SD, n is the total number of analyzed Golgi bodies from 2 independent experiments including 2 biological replicates each. Source data are provided as a Source Data file. (J) MNS3-GFP was transiently co-expressed with GnTI-mRFP or MNS1-mRFP in *N. benthamiana* leaves and purified using GFP-Trap beads. The MNS3-GFP bait and co-purified proteins were analyzed by immunoblotting with anti-GFP and anti-mRFP antibodies. “Input” denotes total protein extracts before incubation with GFP-coupled beads; “bound” denotes the immunoprecipitated fraction eluted from GFP beads. (K) Surface interaction model displaying a possible scenario of how the three Golgi enzymes GnTI, MNS1 and MNS3 may interact within a complex. The calculated FRET efficiencies (E) are as follows: MNS3-GFP/GnTI-mRFP (E=16.6%), MNS3-GFP/MNS1-mRFP (E=7.4%) and GnTI-GFP/MNS1-mRFP[24] (E=11.3%). The model was generated with the Swiss Pdb Viewer. Source data are provided as a Source Data file.

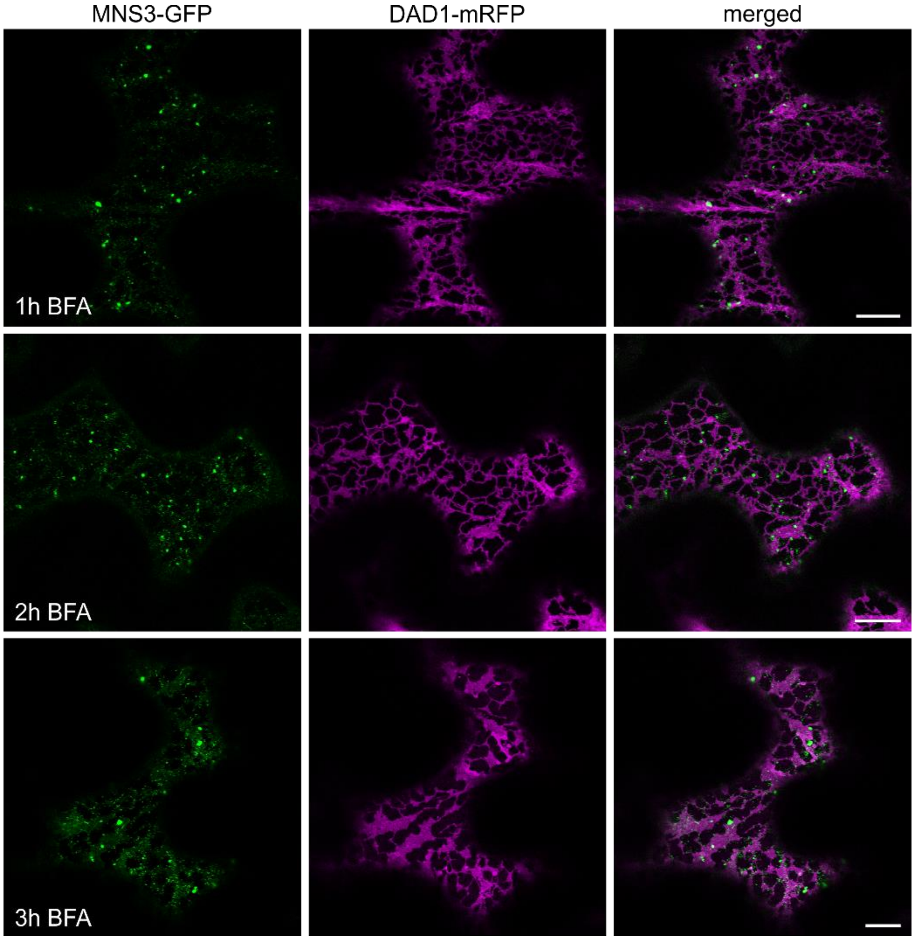

Supplementary Figure 8. BFA treatment of MNS3-GFP co-expressed with an ER marker protein. Confocal images showing a time-course BFA treatment of *N. benthamiana* leaf epidermal cells transiently expressing MNS3-GFP (green) with the ER-resident protein DAD1[76] fused to mRFP (magenta). Images were acquired 2 dpi. Scale bars = 10 µm.

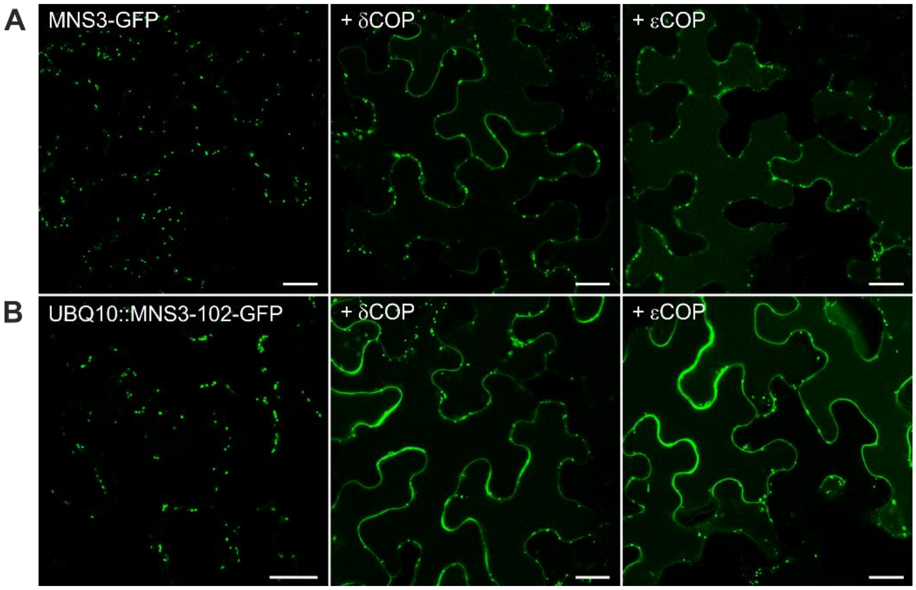

Supplementary Figure 9. Knockdown of the coatomer subunits δCOP or εCOP results in mislocalization of Golgi-resident MNS3-GFP. "+δCOP" indicates co-infiltration with the *N. benthamiana* δCOP RNAi construct. "+εCOP" indicates co-infiltration with the *N. benthamiana* εCOP RNAi construct. (A) Confocal images showing the expression of MNS3-GFP in the absence or presence of the stated RNAi construct. (B) Confocal images showing the expression of UBQ10::MNS3-102-GFP in the absence or presence of the stated RNAi construct. Scale bars = 20 µm.

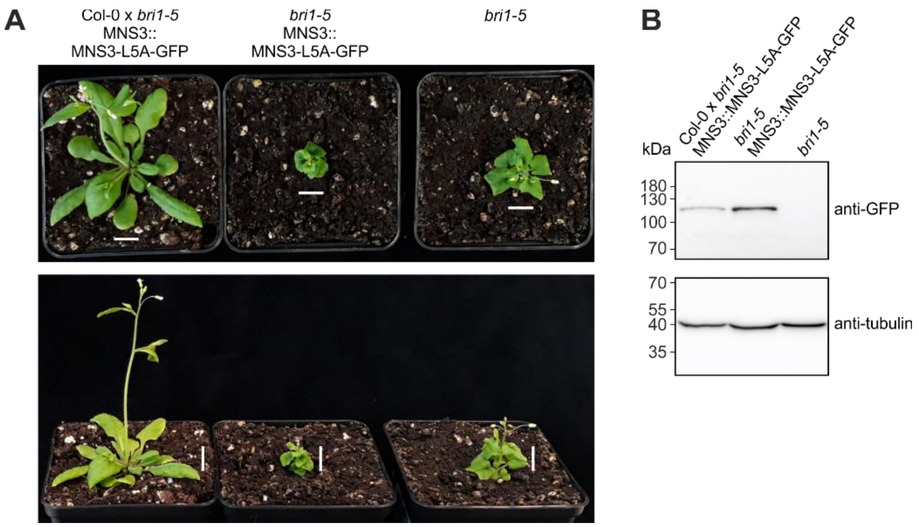

Supplementary Figure 10. Phenotype of backcrossed *bri1-5* expressing MNS3::MNS3-L5A-GFP. (A) Phenotypic comparison and (B) immunoblot analysis of transgenic Arabidopsis *bri1-5* MNS3::MNS3-L5A-GFP backcrossed with Col-0 wildtype, *bri1-5* MNS3::MNS3-L5A-GFP and *bri1-5* plants (22-days old). Scale bars = 1 cm. (B) Protein extracts from leaves were analyzed with antibodies against GFP and tubulin, which was used as a control. The backcrossed Col-0 *bri1-5* MNS3::MNS3-L5A-GFP line shows a weaker GFP signal due to its heterozygous state when compared to the homozygous *bri1-5* MNS3::MNS3-L5A-GFP line. Source data are provided as a Source Data file.

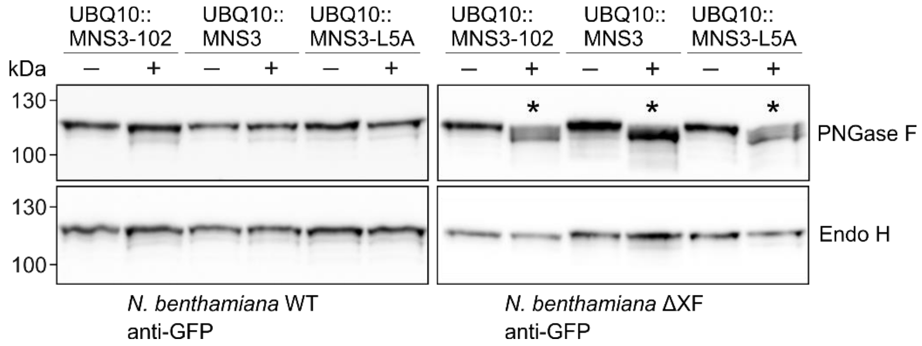

Supplementary Figure 11. MNS3 fusion proteins carry Golgi-processed complex N-glycans with core α1,3-fucose. Endo H and PNGase F digestions of crude protein extracts from *N. benthamiana* wildtype (WT) and ΔXF plants with reduced core α1,3-fucosyltransferase activity, each expressing either wildtype UBQ10::MNS3-GFP, mutated UBQ10::MNS3-L5A-GFP or truncated UBQ10::MNS3-102-GFP. Proteins were separated by SDS-PAGE and immunoblots were probed with an anti-GFP antibody. Mobility shifts are marked with an asterisk. Source data are provided as a Source Data file.

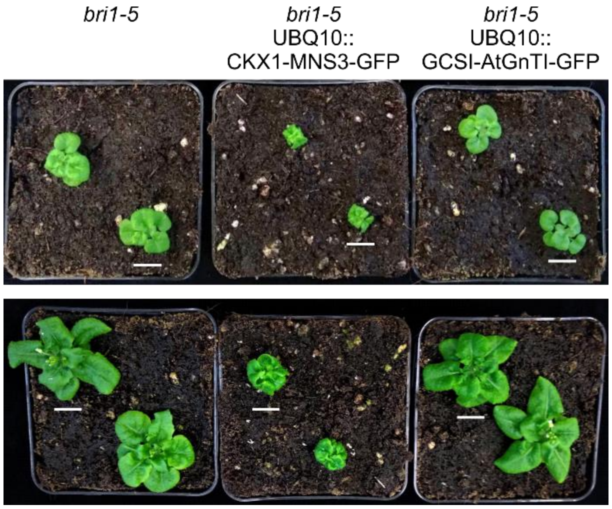

Supplementary Figure 12. ER retention of MNS3 enhances the *bri1-5* phenotype. Phenotypes of Arabidopsis *bri1-5* and transgenic *bri1-5* plants expressing either UBQ10::CKX1-MNS3-GFP or UBQ10::GCSI-AtGnTI-GFP. Images of 22-days-old (upper panel) and 30-days-old (lower panel) soil-grown plants are shown. Scale bars = 1 cm.

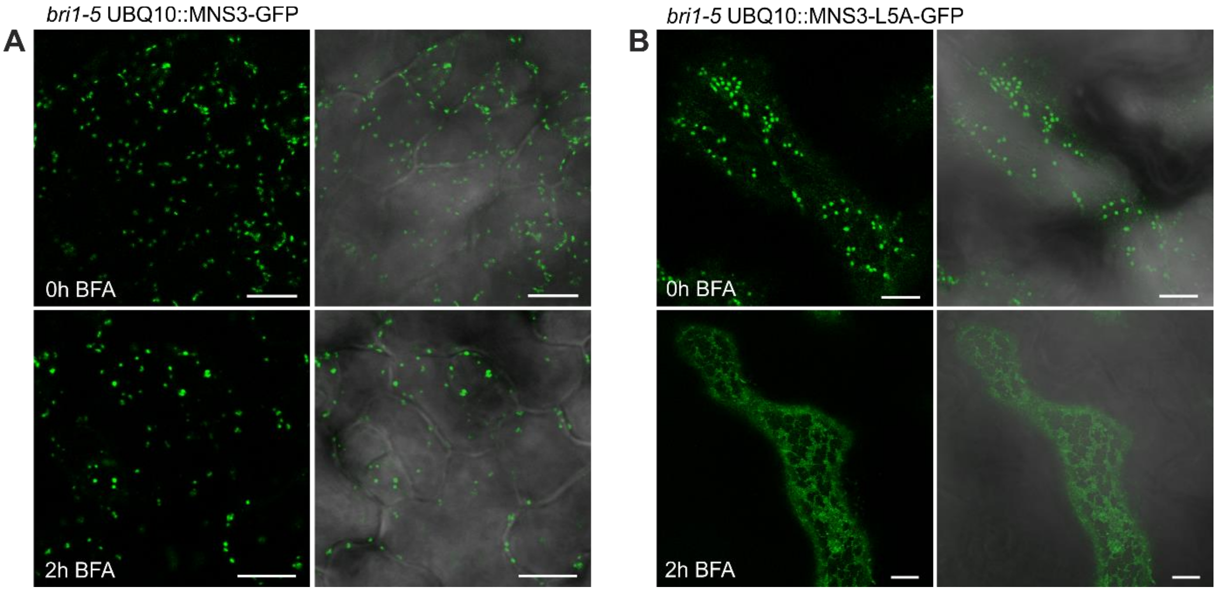

Supplementary Figure 13. The N-terminal cytoplasmic leucine residue is essential for the Golgi retention of MNS3. Confocal images of leaves from transgenic *bri1-5* seedlings expressing either (A) UBQ10::MNS3-GFP or (B) mutated UBQ10::MNS3-L5A-GFP before and after BFA treatment. Seedlings were 8 days old. Scale bars = 10 µm.

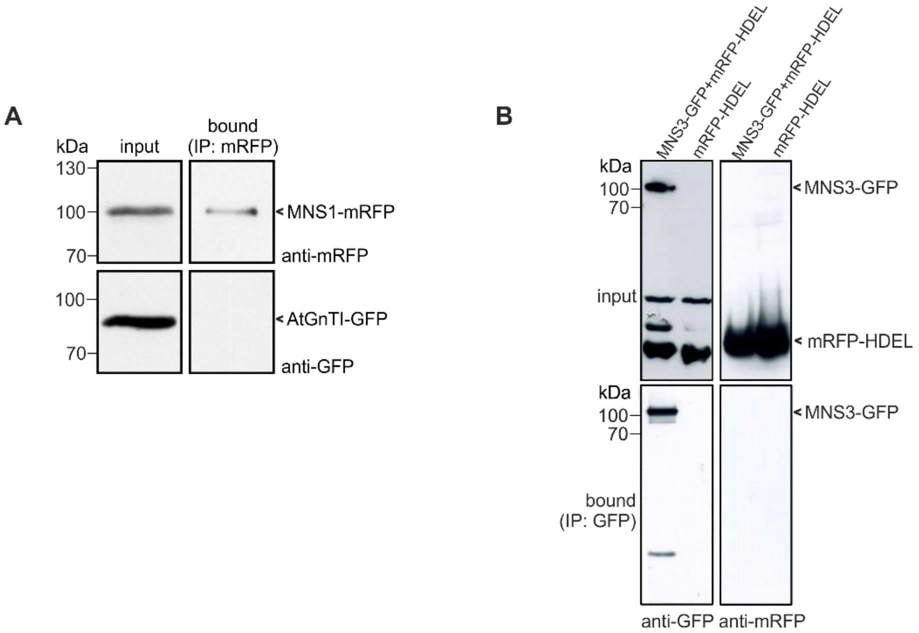

Supplementary Figure 14. Negative control for GFP- and mRFP-Trap purification system. Fusion proteins were transiently co-expressed in *N. benthamiana* leaves and protein extracts were subjected to a one-step purification system (GFP- or mRFP-Trap, Chromotek). (A) Co-expression of MNS1-mRFP and UBQ10::GCSI-AtGnTI-GFP. MNS1-mRFP was purified using mRFP-Trap beads. The mRFP bait and co-purified proteins were analyzed by immunoblotting with antibodies against GFP and mRFP. (B) Expression of MNS3-GFP with mRFP-HDEL or mRFP-HDEL alone. MNS3-GFP was purified using GFP-Trap beads. The GFP bait and co-purified proteins were analyzed by immunoblotting with antibodies against GFP and mRFP. "Input" denotes total protein extracts before incubation with GFP- or mRFP-coupled beads; "bound" denotes the immunoprecipitated fraction. Source data are provided as a Source Data file.

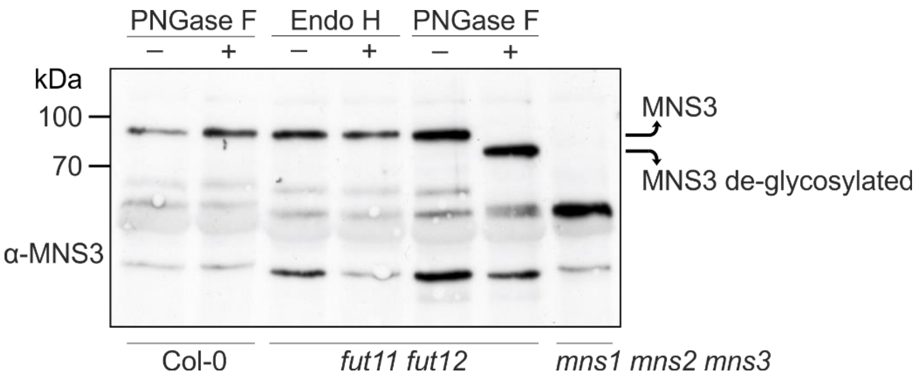

Supplementary Figure 15. Validation of MNS3 antibody specificity. Endo H and PNGase F digestion of crude protein extracts from Arabidopsis wildtype (Col-0) and mutant plants lacking core α1,3-fucosyltransferase activity (*fut11 fut12*). Proteins were separated by SDS-PAGE and blots were probed with an anti-MNS3 antibody. Antibody specificity was tested by probing crude protein extracts from Arabidopsis triple knockout plants deficient in MNS1, MNS2 and MNS3 (*mns1 mns2 mns3*). No cross-reactivity against endogenous Arabidopsis MNS1/MNS2 was observed. Source data are provided as a Source Data file.

Supplementary Table 1. List of oligonucleotide sequences

Name	5'-3'
At1g30000_12F	tataTCTAGAATGTCGAAATCTCTACCATATTCAG
At1g30000_30F	TATAGGTACCTTCTCTTACATTGGACGAGTACAAG
At1g30000_31R	TATAAGATCTGGTGTTTCTTCTTATTGGTAATG
At1g30000_32R	GAGATTTCGACATTCTAGATACCCCCGTTCTAATT
At1g30000_33F	ATCTAGAATGTCGAAATCTCTACCATATTCAG
At1g30000_34F	GGACAATTAAGAGGATCCAGTTCCACTAATGGCTC
At1g30000_35R	CTGGATCCTCTTAATTGTCCTTCGTCAGGTG
At1g30000_37F	tataTCTAGAATGTCTCCTTTAAAGGTTTTTTCT
At1g30000_38R	tataGGATCCTCTTAATTGTCCTTCGTCAGGTGA
At1g30000_40F	TATAACTAGTATGTCGAAATCTCTACCATATTCAG
At1g30000_41F	tataTCTAGAATGCATTACGACAATGCCAAGTTCA
At1g30000_42F	tataTCTAGAATGGTTAAAGATATTCATTACGAC
At1g30000_43F	tataTCTAGAATGCTACCATATTCAGTTAAAGATA
At1g30000_44F	tataTCTAGAATGCTAGCATATTCAGTTAAAGATA
At1g30000_45F	tataTCTAGAATGCTACCAGCTTCAGTTAAAGATA
At1g30000_46F	tataTCTAGAATGGCACCATATTCAGTTAAAGATA
At1g30000_47F	TATATCTAGAATGTCGAAATCTGCACCATATTCAGTTAAA
At1g51590_27F	TATAGGTACCGGTTGCTTTTCATCAATCTACCTAA
At1g51590_28R	TATACTAGATTCTCAACCCACTCAACAAAAAC
At1g51590_33F	TATAACTAGTATGGCGAGAAGTAGATCGATTAG
At1g51590_41F	TATAAGATCTGAAGCAAGTACCTTCCGGAGGTTG
GFP_12R	ATATAGATCTTCTGTATAGTTCATCCATGCCAT
SYP31_1F	TACTCTCGAAATAAAGCTCATCATCTG
SYP31_2R	ACTTTGTTTGCTTTGAATTTTTACTTGA
SYP31_3F	TATATCTAGAGGCTCGACGTTCAGAGATCGGAC
SYP31_4R	TATATCTAGATTAAGCCACAAAGAAGAGGAAAACA
GCSI_7F	TATATCTAGAATGACCGGAGCTAGCCGTCGGAGC
AthGnTI_9R	TATAAGATCTGGAATTTCGAATTCCAAGCTGC
CKX1_5F	TATATCTAGAATGGGATTGACCTCATCCTTACGG
CKX1_6R	TATAGGATCCGTCCTTGGCCACATTGTGGACATCG
