# Supplementary material for: A signal motif retains Arabidopsis ER-α-mannosidase I in the cis-Golgi and prevents enhanced glycoprotein ERAD
Source: Nat Commun. 2019 Aug 16;10:3701. doi: 10.1038/s41467-019-11686-9 (PMC6697737; doi:10.1038/s41467-019-11686-9)
Supplement: Supplementary file 3 — Reporting Summary [file 41467_2019_11686_MOESM3_ESM.pdf]

natureresearch

Corresponding author(s): Jennifer Schoberer

Reporting Summary

Nature Research wishes to improve the reproducibility of the work that we publish. This form provides structure for consistency and transparency in reporting. For further information on Nature Research policies, see Authors & Referees and the Editorial Policy Checklist.

Statistical parameters

When statistical analyses are reported, confirm that the following items are present in the relevant location (e.g. figure legend, table legend, main text, or Methods section).

n/a	Confirmed	
☐	☒	The exact sample size (n) for each experimental group/condition, given as a discrete number and unit of measurement
☐	☒	An indication of whether measurements were taken from distinct samples or whether the same sample was measured repeatedly
☐	☒	The statistical test(s) used AND whether they are one- or two-sided<br>*Only common tests should be described solely by name; describe more complex techniques in the Methods section.*
☒	☐	A description of all covariates tested
☒	☐	A description of any assumptions or corrections, such as tests of normality and adjustment for multiple comparisons
☐	☒	A full description of the statistics including central tendency (e.g. means) or other basic estimates (e.g. regression coefficient) AND variation (e.g. standard deviation) or associated estimates of uncertainty (e.g. confidence intervals)
☐	☒	For null hypothesis testing, the test statistic (e.g. F, t, r) with confidence intervals, effect sizes, degrees of freedom and P value noted<br>*Give P values as exact values whenever suitable.*
☒	☐	For Bayesian analysis, information on the choice of priors and Markov chain Monte Carlo settings
☒	☐	For hierarchical and complex designs, identification of the appropriate level for tests and full reporting of outcomes
☐	☒	Estimates of effect sizes (e.g. Cohen's d, Pearson's r), indicating how they were calculated
☒	☐	Clearly defined error bars<br>*State explicitly what error bars represent (e.g. SD, SE, CI)*

Our web collection on statistics for biologists may be useful.

Software and code

Policy information about availability of computer code

Data collection	Confocal images were acquired with commercial, built-in softwares of Zeiss (Zen blue/black, LSM500), Leica (LAS AF, LAS X) or Nikon (EZ-C1). FRET-FLIM images were acquired using the Becker&Hickl TCSPC PC module SPC-830.
Data analysis	FRET-FLIM images were analyzed using the Becker&Hickl SPCImage software v.9.3. For colocalization analysis, the Pearson's correlation coefficient (using Costes automatic thresholding) was calculated using the IMAGEJ (version 1.46 m) plug-in JACOP (Bolte and Cordelières, 2006). Statistical analyses were performed in Microsoft Excel using a two-tailed Student’s t-test for the comparison of two samples, assuming equal variances.

For manuscripts utilizing custom algorithms or software that are central to the research but not yet described in published literature, software must be made available to editors/reviewers upon request. We strongly encourage code deposition in a community repository (e.g. GitHub). See the Nature Research guidelines for submitting code & software for further information.

Data

Policy information about availability of data

All manuscripts must include a data availability statement. This statement should provide the following information, where applicable:

- Accession codes, unique identifiers, or web links for publicly available datasets
- A list of figures that have associated raw data
- A description of any restrictions on data availability

The authors declare that all relevant data generated or analyzed during this study are included in this article and its supplementary information file. The source data underlying Figures 2C, 5E-F, 6C, 9C, Supplementary Figures 3B, 4, 6B, 7, 10B, 11, 14 and 15, and co-localization analyses are provided in the Source Data file. All other data that support the presented findings are available from the corresponding author upon reasonable request.

Field-specific reporting

Please select the best fit for your research. If you are not sure, read the appropriate sections before making your selection.

☒ Life sciences ☐ Behavioural & social sciences ☐ Ecological, evolutionary & environmental sciences

For a reference copy of the document with all sections, see nature.com/authors/policies/ReportingSummary-flat.pdf

Life sciences study design

All studies must disclose on these points even when the disclosure is negative.

Sample size	No statistical methods were used to pre-determine sample size. For FRET-FLIM or colocalization analyses, we opted for a sample size that has proven to be reasonable in previous, successful experiments with a similar setup.
Data exclusions	For colocalization analyzes, no data were excluded. For FRET-FLIM analysis, data were excluded prior to analysis if photon counts were too low or there was a z-drift of the focal plane.
Replication	In general, experiments (confocal, western blot, treatments etc) were repeated at least 3 times. Confocal data were acquired from 2 biological replicates (plants) per session. For colocalization analyses, data were collected from 2 biological replicates (plants) from 1 infiltration event per expressed combination. For FRET-FLIM, data for the donor and donor-acceptor combinations, respectively, were collected from 2 independent infiltration events, each including 2 biological replicates (plants).
Randomization	For confocal imaging, cells were selected randomly for each sample throughout the slide and representative cells are shown in the manuscript. Depending on protein expression levels or time intervals of drug treatments, 20 to 200 images were acquired per expressed protein or combination in each confocal session.
Blinding	Blinding is not relevant to this study, as protein samples or fluorescent protein fusions were not required to be allocated into experimental groups, and no animals or human research participants are involved in this study.

Reporting for specific materials, systems and methods

Materials & experimental systems

n/a	Involved in the study	
☐	☒	Unique biological materials
☐	☒	Antibodies
☒	☐	Eukaryotic cell lines
☒	☐	Palaeontology
☒	☐	Animals and other organisms
☒	☐	Human research participants

Methods

n/a	Involved in the study	
☒	☐	ChIP-seq
☒	☐	Flow cytometry
☒	☐	MRI-based neuroimaging

Unique biological materials

Policy information about availability of materials

Obtaining unique materials	Any plant mutants or transgenic lines that have been generated in this study are available from the corresponding author upon reasonable request.

Antibodies

Antibodies used

Except for the MNS3 antibody, all used antibodies are commercially available. The MNS3 antibody was generated specifically for this study. For details about generation, please see the materials & methods section "Antibodies" in the main body of the manuscript.

Primary antibodies:
GFP antibody: from Roche (No. 11814460001), mouse monoclonal - mixture of IgG1κ clones 7.1 and 13.1. Dilution 1:2,000.
RFP antibody: from Chromotek (6G6), mouse monoclonal IgG2c. Dilution 1:2,000.
MNS3 antibody: custom-made as described in the material & methods section, produced in rabbit. Dilution 1:2,000.
alpha-tubulin antibody: from Sigma (T6074), mouse monoclonal IgG1- clone B-5-1-2, purified immunoglobulin. Dilution 1:5,000.
Secondary antibodies:
Anti-Mouse IgG (whole molecule)–Peroxidase antibody: from Sigma (A9044), produced in rabbit. Dilution 1:10,000.
Anti-Rabbit IgG (whole molecule)–Peroxidase antibody: from Sigma (A0545), produced in goat. Dilution 1:100,000.

Lot numbers are not given for the commercially available antibodies as these are routinely aliquoted in our lab as recommended by the manufacturer to prevent freeze-thaw damage and multiple lots of the antibodies have been used in our lab in several different (published) studies over many years and the obtained results were always highly consistent.

Validation

Commercial antibodies have been verified by the manufacturers as shown on their websites. Briefly, the GFP antibody from Roche is a monoclonal antibody for the detection of GFP or GFP fusions using immunoprecipitation, western blots or immunostaining (according to the manufacturer). The RFP antibody from Chromotek was tested on mRFP, mCherry, mPlum, mOrange, mRFPruby, DsRed and is only applicable for western blots (according to the manufacturer). The secondary rabbit anti-mouse IgG (whole molecule)–peroxidase antibody has been used in western blotting , immunohistochemistry and protein pin array assay. The secondary goat anti-rabbit IgG (whole molecule)–peroxidase antibody has been used in western blotting , immunofluorescence staining, immunochemistry and immunoprecipitations. The alpha-tubulin antibody from Sigma is suitable for immunocytochemistry, immunoprecipitation, microarray, and western blotting.
Regarding the custom-made MNS3 antibody, the specificity of the antibody was tested in immunoblotting studies with recombinant MNS3 produced as described (Liebminger et al., Plant Cell 2009) and protein extracts of Arabidopsis wildtype (Col-0) and mns1 mns2 mns3 triple knockout plants lacking the MNS3 protein (Supplementary Figure 15). No cross-reactivity against endogenous Arabidopsis MNS1/MNS2 was observed.
